# An Educational Digital Tool to Improve the Implementation of Switching to a Biosimilar (Rapid Switch Trainer): Tool Development and Validation Study

**DOI:** 10.2196/56553

**Published:** 2024-11-21

**Authors:** Carlos Marras, María Labarga, Daniel Ginard, Jose Manuel Carrascosa, Alejandro Escudero-Contreras, Eduardo Collantes-Estevez, Fernando de Mora, Tamara Robles, Elisa Romero, Rafael Martínez

**Affiliations:** 1 Hospital Clínico Universitario Virgen de la Arrixaca, Academia Medicina de Murcia, Servicio Murciano de Salud, Ctra. Madrid-Cartagena, s/n, El Palmar, 30120, Spain, 34 968 36 95 00; 2 Medical Department, Sandoz Farmacéutica, Madrid, Spain; 3 Hospital Universitario Son Espases, Servicio Aparato Digestivo/Fundación Instituto de Investigación Sanitaria Islas Baleares, Palma de Mallorca, Spain; 4 Hospital Universitari Germans Trias i Pujol, Institute for Health Science Research Germans Trias i Pujol, Badalona, Spain; 5 Hospital Universitario Reina Sofía, Instituto Maimónides de Investigación Biomédica de Córdoba), Universidad de Córdoba, Córdoba, Spain; 6 Pharmacology Department, Universitat Autónoma de Barcelona, Barcelona, Spain

**Keywords:** consumer health information, treatment switching, biosimilar pharmaceuticals, immune-mediated diseases, education, qualitative research, training, nocebo, digital tool, implementation

## Abstract

**Background:**

Switching to biosimilars is an effective and safe practice in treating inflammatory diseases; however, a nocebo effect may arise as a result of the way in which the switch is communicated to a given patient.

**Objective:**

We aimed to design a gaming-based digital educational tool (including a discussion algorithm) to support the training of health care professionals in efficiently communicating the switch to biosimilars, minimizing the generation of a nocebo effect and thus serving as an implementation strategy for the recommended switch.

**Methods:**

The tool was developed based on interviews and focus group discussions with key stakeholders, both patients and health care professionals. Messages likely to either generate trust or to trigger a nocebo effect were generated on the basis of the interviews and focus group discussions.

**Results:**

A total 7 clinicians and 4 nurses specializing in rheumatology, gastroenterology, and dermatology, with balanced levels of responsibility and experience, as well as balance between geographic regions, participated in the structured direct interviews and provided a list of arguments they commonly used, or saw used, to justify the switching, and objections given by the patients they attended. Patients with immune-mediated inflammatory diseases who were taking biologic drugs with (n=4) and without (n=5) experience in switching attended the focus groups and interviews. Major topics of discussion were the reason for the change, the nature of biosimilars, and their quality, safety, efficacy, and cost. Based on these discussions, a list of objections and of potential arguments was produced. Patients and health care professionals rated the arguments for their potential to evoke trust or a nocebo effect. Two sets of arguments, related to savings and sustainability, showed discrepant ratings between patients and health care professionals. Objections and arguments were organized by categories and incorporated into the tool as algorithms. The educators then developed additional arguments (with inadequate answers) to complement the valid ones worked on in the focus groups. The tool was then developed as a collection of clinical situations or vignettes that appear randomly to the user, who then has to choose an argument to counteract the given objections. After each interaction, the tool provides feedback. The tool was further supported by accredited medical training on biosimilars and switching.

**Conclusions:**

We have developed a digital training tool to improve communication on switching to biosimilars in the clinic and prevent a nocebo effect based on broad and in-depth experiences of patients and health care professionals. The validation of this implementation strategy is ongoing.

## Introduction

A biosimilar is a biologic medicine highly similar to an original, already approved biologic medicine (the reference medicine) [1]. It has been more than 15 years since the first biosimilar was authorized by the European Medicines Agency (EMA), and the evidence supporting the benefit of biosimilar use, including the practice of switching, is considerable [2-4]. Switching has been verified by regulatory agencies as a safe practice; biosimilars and reference molecules are considered interchangeable [1,5]. Biosimilars have the potential to increase competition, lower costs, and consequently foster increased patient access to biologic medications [6-8]. However, the expected uptake has been limited and is unequal across countries [9,10]. While the EMA and other agencies have addressed all concerns raised regarding pharmaceutical quality, safety (especially immunogenicity), efficacy , and interchangeability with the reference product to the point that interchangeability is now regarded as a scientific concept [5,9,11-13], issues with communication between physicians and patients have scarcely been addressed. Indeed, miscommunication, or biased communication, underlies the suboptimal use of biosimilars and the presence of nocebo effects in patients. Yet learned societies and regulators have taken little action to counteract these deleterious consequences [9,14]. Moreover, after the launch of the first biosimilars, we have all witnessed misinformation, whether intentional or not [15].

The physician acceptance of biosimilars for inflammatory diseases is still at stake, with a vast majority of US physicians, including rheumatologists, dermatologists, and gastroenterologists, not willing to switch stable patients to a biosimilar [16], over 95% of German rheumatologists preferring to prescribe an originator biologic rather than a biosimilar as first- or second-line therapy if unrestricted [17], and French rheumatologists being favorable toward the implementation of biosimilars, but very few actually switching [18]. In Spain, a 2021 survey of multiple specialists found gaps in knowledge about biosimilars and unclear policies and practices, although the general perception and attitude seemed positive [19]. Overall, a systematic review of studies exploring the attitudes of physicians showed that 65% to 67% have concerns about the use of biosimilars in patients [20].

Negative responses to inert interventions in clinical practice stemming from patients’ negative expectations, in other words, the nocebo effect, may foster loss of efficacy, lack of adherence, or the occurrence of adverse events when switching from a reference drug to a biosimilar [21-24]. Negative expectations of biosimilar drugs [13] may arise from the patient’s beliefs or may be induced by a health care professional [23]. Poor communication may underlie some patients’ reluctance to accept biosimilars, despite being in favor of statements about biosimilars’ safety and efficacy and about switching [17,25-27]. Anxiety related to changes for reasons other than clinical status [26], as well as fear of negative effects, a lack of efficacy, a loss of control, or an increase in side effects [17,26,28], all pave the way for a nocebo effect [28,29]. Clearly, if the physician has gaps in knowledge or biased knowledge, despite a positive attitude toward biosimilars, the chances of a failed switch increase. Therefore, adequate patient communication can help minimize the nocebo effect’s impact.

The difficulty with proper communication lies in the fact that physicians, due to time constraints, are not able to engage in longer patient-centered interactions, which are key for the acceptance of biosimilars [25,30]. To increase the willingness of health care professionals to use biosimilars successfully, some have proposed improving the communication between companies and physicians [31], including easier access to balanced educational materials about biosimilars with consistent, fair, and positive messages about their value to counter misinformation [15]. However, in light of the lack of experience with such tools, there is only scarce information on their efficacy.

The difficulty lies in the implementation of education and communication strategies between the physician and the patient rather than among professionals, for which best practice examples have been reported [32-34]. Educational material has been created to aid in communication between health care professionals and patients about biosimilars [35]. Some of this material includes messages addressing the adaptation to a new delivery device and framing to help counteract the nocebo effect [29,36-39]. However, such material was created to guide, but not to train, health care professionals, who, at the end of the day, base their effort on voluntary work and lack the time to use the material effectively.

In particular, in the context of time constraints, we propose to optimize clinician-patient communication by designing a digital tool that would train clinicians to communicate the originator-to-biosimilar switch to patients properly. The objective was thus to design and implement a gaming-based communication digital tool (a discussion algorithm) to support medical experts in communicating about the switch to biosimilars in a short time, and consequently to reduce the nocebo effect in patients.

## Methods

### Overview

The tool was developed and tested between December 2020 and May 2023 in four phases: (1) interviews with health care professionals to identify narratives; (2) interviews with patients who had experienced a switch, a focus group with patients who had not experienced a switch, and focus groups with clinicians and nurses to validate and grade the messages; (3) development of a digital training tool that was tested by a scientific committee; and (4) validation and implementation. This last phase is still ongoing.

The scientific committee acting during phase 3 comprised a rheumatologist as coordinator (CM), a dermatologist (JMC), a gastroenterologist (DG), and an additional rheumatologist (AE-C), who supervised the whole process of validating and endorsing the digital tool.

Participants in all project phases were selected by purposive sampling and recruited from among the authors’ contacts, who represent a broad range of the professionals the study wanted to represent.

### Phase 1: Interviews With Health Care Professionals

We conducted 11 structured direct interviews by telephone with potential users of the digital training tool, that is, health care professionals in rheumatology, dermatology, and gastroenterology, including both clinicians and nurses. The outline of the interviews included questions on their experience in the use of biosimilars and switching, the main reasons for switching, how they explained to the patient the biosimilar concept and why they had to switch, how they felt about switching, whether they adapted the message to a given patient, how long the conversations were, whether they used the terms “economic” or “efficient” and their emotional charge for them and their patients, whether the patients understood, and what their concerns were (the questions are listed in Multimedia Appendix 1). They were also asked about the nocebo effect, resistance to change, and questions or objections the patients may have had about efficacy, safety, cost, efficiency, responsibility for the change, the reason for the change, avoidance, and delivery. The interviews, led by an external expert, were recorded and analyzed inductively.

Following the information obtained in phase 1, validated messages were generated according to different scientific references and tested in phase 2.

### Phase 2a: Focus Group of Patients

Patients with immune-mediated inflammatory diseases taking biologics without having experienced a switch from a reference product to a biosimilar were invited to a focus group, and patients who had experienced a switch were invited to individual interviews. Nephila, an agency specializing in qualitative research and communication, recruited the participants from patients’ associations and through their treating physicians; all participants signed informed consent forms. The sessions were held virtually in June 2021, lasting between 90 and 100 minutes, and were conducted by external researchers with experience in group moderation. Scripts were structured into thematic blocks based on the validated messages from phase 1. A web-based tool was used to dynamize the focus groups and obtain a sense of quantitativeness in the answers [40].

### Phase 2b: Focus Groups of Health Care Professionals

Two focus groups, one with clinicians (October 2021) and another with nurses (November 2021), were held to outline the tool’s content and format. The messages previously tested in the patient focus groups and interviews were compiled into a content proposal, developed in two blocks: (1) basic concepts in the therapeutic switch and (2) objections by theme, including the concept of biosimilars; their efficacy, safety, and quality; administration and medical devices; reasons for switching; efficiency; and cost. In each block, different questions and answers were proposed, with the messages tested on patients and rated positively, average, or not positively. Before both sessions, all attendees could individually assess each of the questions posed and add comments. In subsequent meetings, the proposed content was validated by evaluating the communication efficacy of the answers. With this, the participants helped develop “vignette” conversations that could be used as objection handlers, that is, that could be used in typical situations in which patients challenge the switch and included typical and not-so-typical responses based on the results of the previous phases. Five patient types were designated: (1) conformists, (2) patients focused on efficacy objections, (3) patients focused on sustainability objections, (4) patients focused on objections related to adverse effects, and (5) patients who mixed all objections. Later, when these types were fully developed, they were presented at random to the users.

### Phase 3: Development of the Digital Training Tool

This phase took place from March 2022 through May 2023. The development team was formed by educators and IT developers with experience in training apps. All the vignettes proposed in the focus groups were converted into “visits” and organized into a grid, which became the core of the digital tool. Each “visit” would present a situation with several potential responses. Each response had a level of efficiency assigned based on the focus group results. Based on all the questions posed by the avatar patient and the responses by the avatar doctor, the system provided feedback on the level of confidence, and the health care professional was also provided with a rationale for the best responses. The digital tool prompted the user to try several scenarios until a certain level of maintained efficacy was reached.

### Ethical Considerations

The Ethics Committee of Research of the Arrixaca Hospital was consulted on the need for approval, and they replied that the project was exempt. The team, nevertheless, followed all applicable rules for good practices, including asking for informed consent to collect information during the sessions and confidentiality rights—names were not disclosed in the reports produced by the agencies conducting the interviews and focus groups. Clinicians and nurses participating in phases 1 and 2 and the scientific committee that assisted in reviewing and validating the tool in phase 3 received payment according to local fair market value commensurate with their contract hours. Patients in phase 2 received a token fee for their help and participation through the Nephila Agency (never directly through Sandoz).

## Results

### Phase 1: Interviews With Health Care Professionals

A total of 7 clinicians and 4 nurses from 7 different regions of Spain were interviewed: 6 worked in rheumatology, 2 in gastroenterology, and 3 in dermatology, with balanced levels of responsibility and experience and balance between regions (Table S1 in Multimedia Appendix 2).

According to their responses, approximately 30% to 40% of the health care professionals’ patients were using biosimilars. When asked how many of these had been switched from the reference drug, the reported percentages ranged from 10% to 85%. The main reason for switching was economic or that the switch was fostered by the region or local regulators. Table S2 in Multimedia Appendix 2 shows a summary of the arguments heard by the health care professionals when introducing a switch to patients. The majority said they adapted the message to the individual patient, but others simply followed the internal protocols and the rationale they had received as guidance. Of note, they said they used 5 to 10 minutes (nurses used even more time) to explain the change. Only 2 referred to educational material to support their conversations—more often, they referred the patient to online information from scientific societies. The heterogeneous perception of the patients’ understanding was also of interest.

As for the objections to switching, Table S3 in Multimedia Appendix 2 groups the perceptions of the health care professionals interviewed. Several interviewees said they had detected a nocebo effect in their patients, mainly in those with previously well-controlled conditions or who did not understand the switch. Physicians also mentioned that if a patient did not want the change, they did not force it—some patients even suggested buying the reference biologic themselves to avoid the change.

In general, the interviewees expressed their interest in a communication aid.

### Phase 2a: Focus Group of Patients

A total of 5 patients without experience in switching participated in the focus group, and 4 patients with experience participated in the interviews. The key results were pooled together (except the experience of the actual switch) and organized into the dimensions of analysis: (1) perception of the communication regarding the switch, and (2) key messages required during this process.

A positive reception of the switch was mentioned in relation to (1) the quality and volume of information received from the specialists; (2) trust in their professionalism and knowledge; (3) the ineffectiveness of or dissatisfaction with the current biologic treatment, meaning that the switch represented an alternative for improvement; (4) certainty of obtaining the same results with both treatments; and (5) a guarantee of a reduction in adverse effects when starting a biosimilar compared to those experienced with the reference. Of note, the first 2 arguments were raised by patients who had already experienced a switch, and the last 3 were arguments in favor of switching among those patients without previous experience. The factors that could explain a negative reaction to the switch were (1) uncertainty about the benefits and impact on disease progression, especially among those satisfied with their biologic treatment; (2) fear of the change being a trial to test the biosimilar drug; and (3) lack of information about the reason for switching, or a perception that the reason was merely economic. Many maintained that the health care professional should have spent more time providing detailed information about this type of medication. As to the content of this information, the patients highlighted possible adverse effects, benefits over the reference, differences between biologics and biosimilars, the administration schedule (eg, storage measures and options in the event of needing to transport the medication), mode of action, possibilities of resuming the previous treatment in case of dissatisfaction with the biosimilar, equivalent efficacy and safety, and comparable quality. Furthermore, the patients expressed that the information should be provided in language adapted to patients’ needs and preferences, preferably in a digital format or on paper for those with difficulty accessing or using communication technologies; they also expressed a preference to receive the information only from their specialist.

Table 1 shows the key messages that obtained a positive reaction.

**Table 1.** Key messages that obtained a positive reaction to a switch according to the discussions and literature. Good and poor response options are presented in their respective columns.

Messages/questions	Good responses	Poor responses
What does biosimilar mean?	“Same active principle”	“Highly similar drug” “Essentially the same” “Same molecule”
What is the efficacy and safety compared to the reference?	“The assessment of the efficacy and safety of the biosimilar follows the same stringent standards of quality, efficacy and safety requirements by the regulatory agencies”	“The efficacy and safety expected for both drugs are the same” “Equivalent effectiveness and comparable safety”
Will this mean a new injectable device?	“Innovation in technology”	“Each manufacturer can innovate and improve the delivery system” “New device”
Why the switch?	“Allow more patients to be treated or increase access to more patients” “Generating savings”	“More efficient than biologics” “Cheaper” “Another brand of the same drug”
What’s the difference between the biosimilar and the reference?	“Being treated with a biosimilar is the same as being treated with the reference medicine in terms of efficacy, quality and safety”	“Highly similar”
How long have they been tested?	“Long track record with biosimilars (15 years) and their approval by agencies such as the European Medicines Agency” “No biosimilar has had to be withdrawn from the market due to efficacy and safety concerns” “European Union supervision”	“The efficacy-safety balance is positive for any approved biologic, and it is not associated with an increased health risk”
What’s the added value?	“It allows more patients access to biological treatments because they are cheaper and encourages competition between companies to innovate in new treatments.” “It allows savings that can be used for other purposes such as increasing the number of patients treated with biologics, paying for other therapies or financing drugs” “In the end, it improves patient access to these treatments and contributes to the efficiency of the National Health System.”	“The innovation of the mode of administration and its personalisation and the savings for the Health System” (without mentioning accessibility and other benefits)

### Phase 2b: Focus Groups of Health Care Professionals

The groups included 7 clinicians and 6 nurses from the departments of rheumatology, gastroenterology, and dermatology. Based their comments, we simplified the messages, changed them slightly to make them more comprehensible, and added more messages. The groups disagreed with the patients’ views on the convenience of including “savings” and “sustainability” as arguments. Finally, they proposed a modular tool with levels of complexity and additional information and introducing gaming aspects to increase engagement. The synthesis of the focus groups was compiled into a grid of potential arguments with grading of their confidence level (in a trust/nocebo scale with 3 traffic light coding levels), feedback on why the confidence was low or high, and optional answers to provide better confidence (an example of the latter is shown in Table S4, Multimedia Appendix 2).

### Phase 3: Development of the Digital Training Tool

In the first step, a training syllabus was developed for the information obtained in the previous steps, and further documentation was used. The educators then developed additional arguments (ie, inadequate answers) to complement the valid ones worked on in the focus groups. These were validated by the scientific committee. In parallel, all the messages (questions and answers) were categorized, tabulated, and coded (with traffic light coding as inadequate, average/neutral, or positive). An example of these steps can be seen in Multimedia Appendix 3 and Multimedia Appendix 4.

In the second step, a mock-up was developed, including modular visits with 3 to 5 questions with answers chosen at random, given that (1) there is always only one optimal answer, (2) all are relevant to the category, (3) the impact on trust or nocebo of the chosen options can be tracked and counted, and (4) a feedback screen is displayed after each decision with a better alternative if applicable. In this version, comments on elements such as errors and wording that needed improvement could be submitted to the developers for fine-tuning.

**Figure 1.**
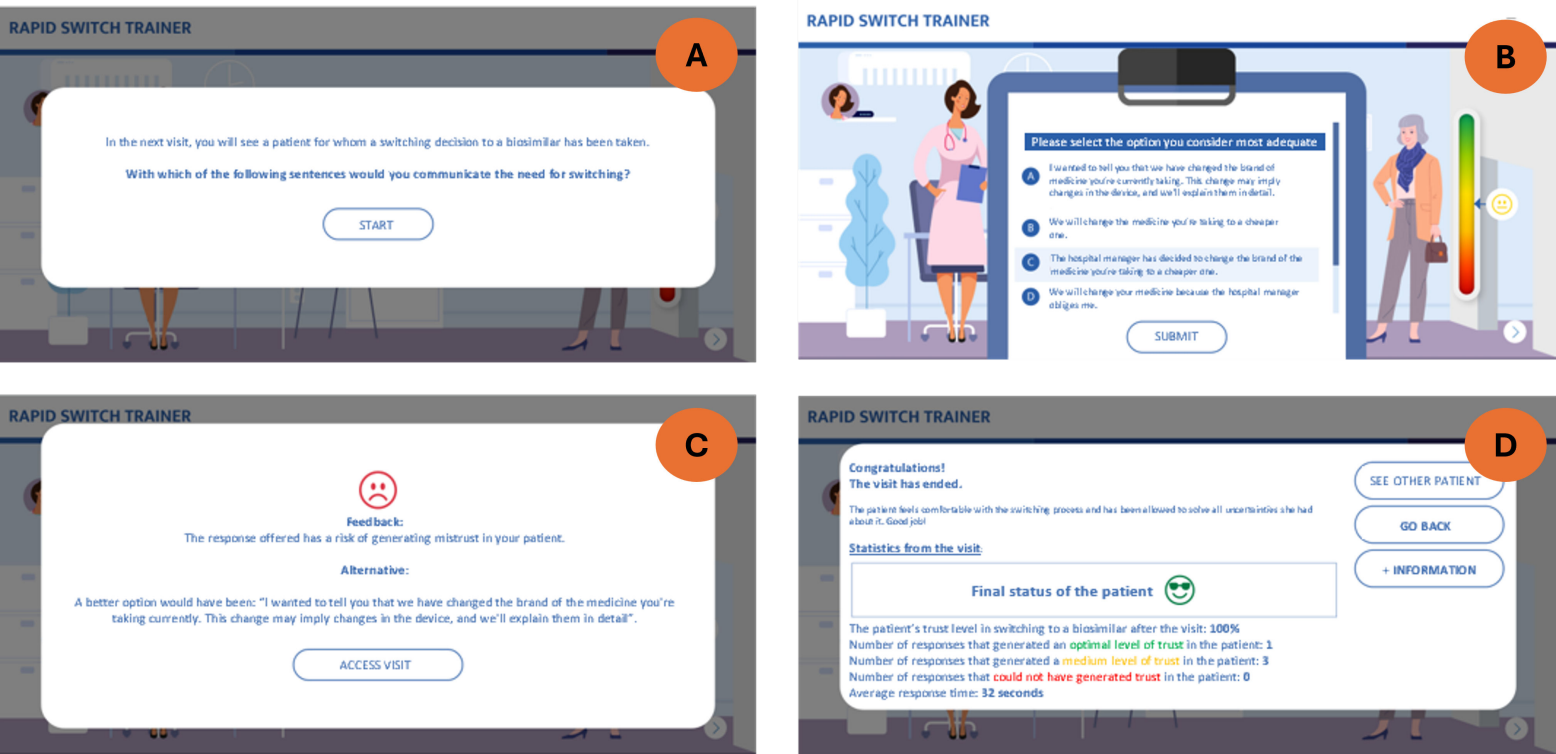
Screenshots of the digital training tool for biosimilars: (A) scenario setting, (B) response options, (C) feedback, and (D) visit statistics.

Next, additional features were implemented: a custom library of arguments and a learning system, different profiles of patients focused on different topics, and educational resources, such as a link to an accredited biosimilar course. Figure 1 depicts sample screens of the digital tool showing the app’s main functionalities. In addition to the interactive vignettes that the doctors used to train on the best responses to random scenarios, the digital tool facilitated access to basic information on the nocebo effect, the rationale for the tool, what a biosimilar is, and the efficacy and safety of biosimilars. Multimedia Appendix 2 contains the technical specifications of the tool, and Multimedia Appendix 4 shows examples of the contents of the tool.

## Discussion

### Principal Findings

Using multiple sources of feedback, including patients, clinicians, nurses, and educators, we have developed a gaming-based training strategy to increase the confidence of health care professionals during challenging situations related to switching from reference biologic drugs to biosimilars. The resulting digital tool has face and content validity, as the vignettes included in the digital tool are based on published research and real situations experienced by patients and health care professionals.

### Comparison to Prior Work

Successful treatment with biosimilars and prevention of nocebo effects heavily rely on patients’ comprehension; thus, when physicians possess a comprehensive understanding of biosimilars and use thoughtful communication strategies, they can effectively support patients who are either starting or transitioning to biosimilars [6].

When a health care professional is confident in their knowledge of a medication, here biosimilars specifically, being aware of the factors that induce bias, that is, using the results of health psychology research, may help reduce the nocebo effect [29,37]. This approach has already enhanced patient-doctor discussions in oncology and is now a daily-life experience during treatment for inflammatory diseases [35].

Health psychology research has confirmed a number of strategies to avoid negative expectations and the nocebo effect. These include balanced information on the risks and benefits of all options, framing (eg, focusing on positive attributes of the medication), transparency, the understandability of the information, shared decision-making, patient empowerment, mode of delivery (verbal or written), being tailored to individual need, and ensuring that the care team speaks with one voice/message [29,36-38,41]. In this sense, the whole multidisciplinary team, of which the hospital pharmacist also forms a part, should be included in the process of switching and provide consistent messages to the patient [42,43]. We cannot forget that patients’ associations should also be included in the debate and that the training should be aligned with their views [40], as they, in the end, are part of the health care system and are involved in aspects of health not covered by the public system. Alignment with them will enhance the one-voice message and help provide understandable information to supplement verbal communication.

Balanced information is thus a critical aspect of reducing the nocebo effect. For biosimilars, consensus-based guidelines are needed; specifically, guidelines are needed that cover preswitch considerations, considerations for each specific drug or class, how to monitor efficacy, and when and to whom to refer patients with challenging arguments [44]. However, these guidelines are complicated to implement in clinical practice. Our digital tool is meant to help with the implementation, but the short arguments allowed by the available consultation time and suggested by our tool should be supported by printed or online information. Both patients and health care professionals in our study identified the lack of needed or preferred information as a source of reluctance during switching, contributing to the nocebo effect. Therefore, the digital tool should be only a starting point to allow for further dialogue, such as telephone calls to address concerns, as proven by a systematic review [36]. Additionally, to increase confidence in biosimilars and avoid the nocebo effect, this tool is supported by accredited medical training on biosimilars and switching, which might also help physician-patient communication.

We hypothesized that scenario-based gaming that presented typical situations and allowed learning about efficient responses to common challenges with feedback and better alternatives, when applicable, would induce efficient behavioral changes in medical communication; we considered that providing answers to challenging situations in switching would be less time consuming and avoid the nocebo effect in patients. The ideal study to test our hypothesis would be an observer-based qualitative study that investigated the use of more- or less-appropriate behaviors (ie, arguments) while measuring the time needed for explanation. An ideal study would also measure the nocebo effect, but this effect cannot be measured. This was, therefore, a challenge, which we tried to address with sufficient face and content validity; we based the vignettes in the digital tool on real situations, with input from patients and health care professionals, and published research. Importantly, we also included nurses, as they are a critical component of the transition to biosimilars, as they lead patient education and are more knowledgeable, in general, about structured and effective communication strategies than doctors [45].

Negative or positive ratings of the arguments were based on the perceptions of patients, both those with and without experience with switching, and of health care professionals with experience in switching. It can be argued that these perceptions were subjective, but confidence is subjective as well, and this is what we wanted to build up. Understanding that “poorly suited” concepts are not necessarily incorrect is important. In fact, many of them are perfectly true; however, they are not as effective in building trust and may contribute to the nocebo effect. It is crucial for the success of the tool to help the health care professional internalize this nuance. It should be noted that the digital tool does not identify right or wrong concepts—implying a binary vision—but, rather, understands that communication moves within a nocebo-to-confidence continuum, which helps reinforce the message and the learning. To achieve this, any tool should explain (1) why each argument is suitable (feedback) and (2) which alternative would be more suitable.

### Strengths and Limitations

First, the participants in the interviews had, in general, broad experience with biosimilars; therefore, the experience of professionals less used to switching might not have been captured as well.

Second, given their complexities, hospitals may have different communication circuits that were not considered in the clinical situations incorporated in the digital tool.

Third, for the interpretation of the results of the qualitative studies, it should be borne in mind that the results should not be extrapolated to all patients under analysis in Spain; first, because of the methodology, and second, because of the small number of patients consulted in both subgroups.

Fourth, the training presented here needs to be proven to be efficacious to build confidence. For this, we have included key performance indicators in the digital tool and will run tests in several centers to validate performance and impact.

Finally, the level of detail of the development process that can be presented in a paper such as this one is limited. Still, the team is open to sharing all documentation and discussing details if needed to ensure that this study is reproducible.

### Future Directions

The tool is being implemented, and we hope to obtain user feedback in the next year, but it is still an ongoing project. If successful, the tool could be customized as doctors gain experience using it (eg, they could add or alter arguments). Fortunately, our contribution will only be temporary; the process will be similar to the one that previously occurred with generic medications. Progressively, and not only based on the growing evidence (from clinical trials and real-world evidence), the acceptance by physicians and patients will increase in initiating and also in switching to biosimilars [46].

### Summary and Conclusion

In summary, in the context of time constraints at clinics and misconceptions about biosimilars, we have developed a digital training tool to improve communication about the switch to biosimilars in the clinic and avoid a nocebo effect based on broad and in-depth experiences of patients and health care professionals; the impact of using the tool is yet to be demonstrated.

## Supplementary material

10.2196/56553 Multimedia Appendix 1 Questions included in the interviews.

10.2196/56553 Multimedia Appendix 2 Supplementary tables and technical specifications.

10.2196/56553 Multimedia Appendix 3 Screenshots of the digital training tool for biosimilars.

10.2196/56553 Multimedia Appendix 4 Examples of rapid switch trainer tool contents.

## Abbreviations

EMA: European Medicines Agency
