# Supplementary material for: An Educational Digital Tool to Improve the Implementation of Switching to a Biosimilar (Rapid Switch Trainer): Tool Development and Validation Study
Source: JMIR Form Res. 2024 Nov 21;8:e56553. doi: 10.2196/56553 (PMC11612528; doi:10.2196/56553)
Supplement: Multimedia Appendix 1 [file formative-v8-e56553-s001.docx]

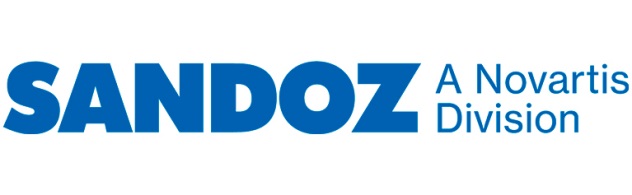

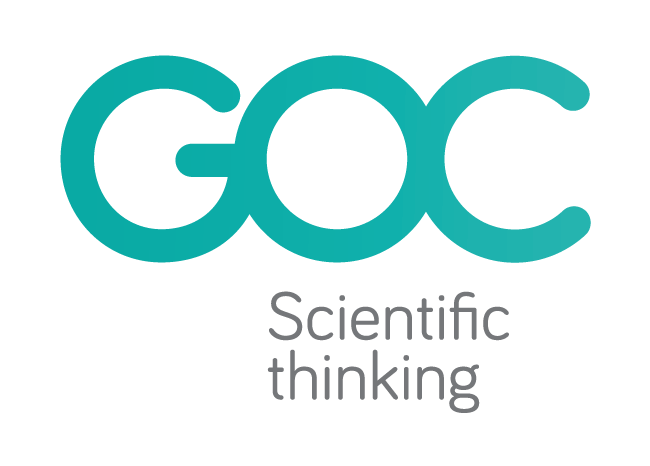
**
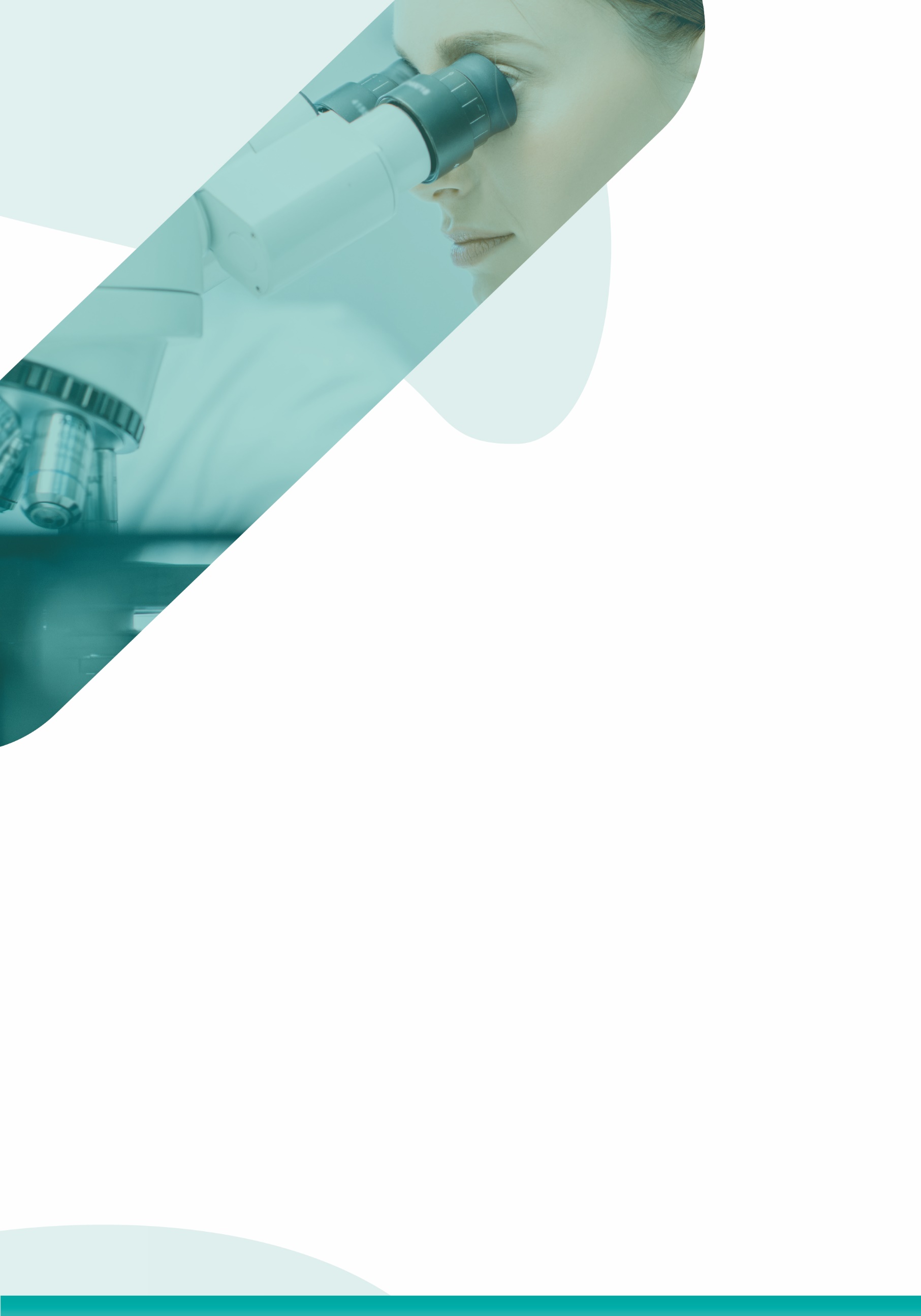
**

**Switching to Biosimilars -**

**Elevator Speech**

**Interview questions for HCPs (Specialists)**

**December 2020**

INDEX

[1. Profile of the health professional 3](#_Toc57365701)

[2. Experience in the use of biosimilars 3](#_Toc57365702)

[3. Practitioner communication with the patient 3](#_Toc57365703)

[4. Patient perception of the therapeutic exchange 4](#_Toc57365704)

[5. Nocebo effect on patients 4](#_Toc57365705)

[6. Key messages to the patient 5](#_Toc57365706)

**Disclaimer**: Interview on biosimilar concept, neither the questions nor the answers should be associated with specific pathology or products. It is not intended to collect clinical information from patients or adverse events.

**Project objective:** The objective of this project is to build a communication tool for therapeutic exchange with the patient focused on efficiency in the shortest possible time in order to reduce the possible nocebo effect on patients.

1. Profile of the health professional
2. Speciality:
3. Position held in the hospital: doctor, head of department, head of section, head of specific consultation, others.
4. Years in the speciality:
5. Years of practice with pathologies requiring biological and biosimilar treatment:
6. Experience in the use of biosimilars
7. Percentage of patients:
   1. Biological reference medicinal product:
   2. Biosimilar medicine:
8. Approximately what percentage of patients have undergone therapeutic switch from the reference biological medicine to the biosimilar?
9. What was your main reason for making the therapeutic switch from the reference biological medicine to the biosimilar?
10. Communication by the professional with the patient
11. Regarding the communication of biosimilars to the patient,
    1. How do you explain what a biosimilar is to patients?
12. In the context of a therapeutic switch from the reference medicinal product to the biosimilar:
    1. Do you explain to the patient that you are switching to the biosimilar?
    2. How do you do it (we are referring to logistical aspects: flow, professionals involved)?
    3. What message do you give to the patient when you communicate the therapeutic exchange? Do you structure the communication in any way?
    4. Are you comfortable communicating the therapeutic exchange? Please elaborate on your answer.
    5. Do you tailor the message according to the patient profile (e.g. age, socio-cultural level, etc.)?
    6. How much time do you devote to communication and do you think it would be necessary to be able to devote more time to it?
    7. Does it explain the reason for the therapeutic exchange?
    8. Do you talk about the biosimilar being a cheaper drug, do you use the term "efficiency" in your message, and is there a positive perception of the word "efficiency" by patients? Please elaborate on your answer.
    9. Do you use any resources (e.g. materials) to do this communication with the patient? If not, would you consider it useful? Digital or physical format?
13. Patient perception of the therapeutic exchange
14. Do you think the patient understands the message conveyed about the biosimilar concept and therapeutic interchange?
15. With regard to the communication of therapeutic exchange what approximate percentage of patients:
16. He receives it well, trusts the treatment and his doctor's recommendation.
17. He is reluctant to change:
    - 1. Why do you think this is perceived as negative?
      2. How do you think this rejection could be minimised?
18. What questions do patients often ask you about the change?
19. Do patients have prior information about biosmilars from sources outside the healthcare sector (e.g. internet, patient associations, etc.)? Do you find more reluctance among these patients?
20. Nocebo effect on patients
21. Do you know the meaning of the nocebo effect? Have you ever detected a nocebo effect in your patients (yes/no)?
22. What do you consider to be the main causes of this nocebo effect?
23. Is there greater resistance to change depending on the patient profile (e.g. socio-cultural factors/age/social networking activity)?
24. Key messages for the patient
25. You can think of some questions or objections that the patient may ask about:

- Effectiveness
- Safety (including immunogenicity)
- Price
- Efficiency
- Responsibility for change
- Reason for change
- Possibility to avoid therapeutic exchange
- Device

1. In all your experience of communication in the therapeutic exchange, which messages do patients receive best and which worst?


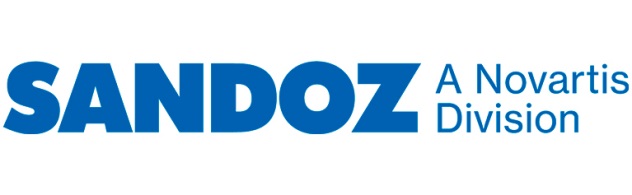

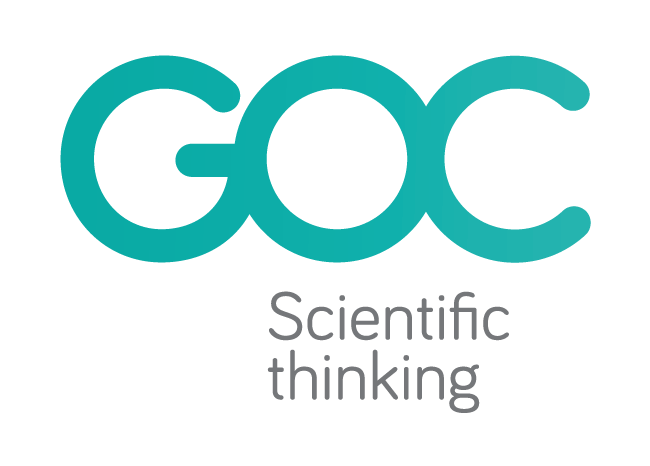
**
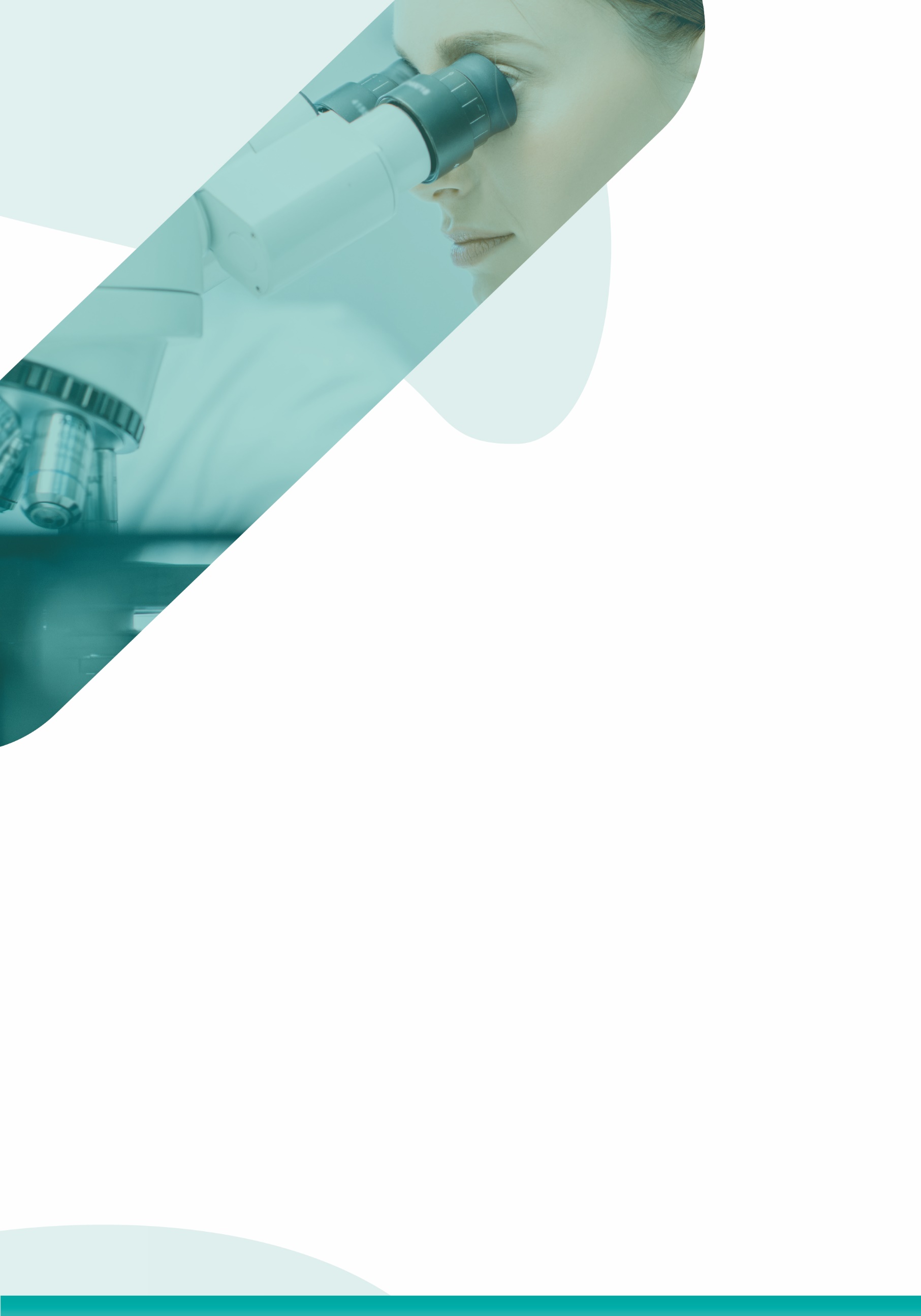
**

**Switching to Biosimilars -**

**Elevator Speech**

**HCP interview questions (Nursing)**

**December 2020**

INDEX

[1. Profile of the health professional 3](#_Toc57365701)

[2. Experience in the use of biosimilars 3](#_Toc57365702)

[3. Practitioner communication with the patient 3](#_Toc57365703)

[4. Patient perception of the therapeutic exchange 4](#_Toc57365704)

[5. Nocebo effect on patients 4](#_Toc57365705)

[6. Key messages to the patient 5](#_Toc57365706)

**Disclaimer**: Interview on biosimilar concept, neither the questions nor the answers should be associated with specific pathology or products. It is not intended to collect clinical information from patients or adverse events.

**Project objective:** The objective of this project is to build a communication tool for therapeutic exchange with the patient focused on efficiency in the shortest possible time in order to reduce the possible nocebo effect on patients.

1. Profile of the health professional
2. Speciality:
3. Position held in the hospital:
4. Years in the speciality:
5. Years of practice with pathologies requiring biological and biosimilar treatment:
6. Experience in the use of biosimilars
7. Percentage of patients:
   1. Biological reference medicinal product:
   2. Biosimilar medicine:
8. Approximately what percentage of patients have undergone therapeutic switch from the reference biological medicine to the biosimilar?
9. Communication by the professional with the patient
10. With regard to the communication of biosimilars to the patient,
    1. How do you explain what a biosimilar is to patients?
11. In the context of a therapeutic switch from the reference medicinal product to the biosimilar:
    1. Do you explain to the patient that the switch to the biosimilar will be made?
    2. How do you do it (we are referring to logistical aspects: flow, professionals involved)?
    3. What message do you give to the patient when you talk about the therapeutic exchange? Do you structure the communication in any way?
    4. Are you comfortable explaining the therapeutic exchange? Please elaborate on your answer.
    5. Do you tailor the message according to the patient profile (e.g. age, socio-cultural level, etc.)?
    6. How much time do you devote to communication and do you think it would be necessary to be able to devote more time to it?
    7. Does it explain the reason for the therapeutic exchange?
    8. Do you talk about the biosimilar being a cheaper drug, do you use the term "efficiency" in your message, and is there a positive perception of the word "efficiency" by patients? Please elaborate on your answer.
    9. Do you use any resources (e.g. materials) to do this communication with the patient? If not, would you consider it useful? Digital or physical format?
12. Patient perception of the therapeutic exchange
13. Do you think that the patient is clear about the concepts of biosimilar and therapeutic interchange before his or her consultation?
14. With regard to the communication of the therapeutic exchange what approximate percentage of patients:
15. He receives it well, trusts the treatment and his doctor's recommendation.
16. He is reluctant to change:
    - 1. Why do you think this is perceived as negative?
      2. How do you think this rejection could be minimised?
17. What questions do patients often ask you about the switch to biosimilars?
18. Do patients have prior information about biosmilars from sources outside the health care setting (e.g. internet, patient associations, etc.) and do you find more reluctance among these patients?
19. Nocebo effect on patients
20. Do you know the meaning of the nocebo effect? Have you ever detected a nocebo effect in your patients (yes/no)?
21. What do you consider to be the main causes of this nocebo effect?
22. Is there greater resistance to change depending on the patient profile (e.g. socio-cultural factors/age/social networking activity)?
23. Key messages for the patient
24. You can think of some questions or objections that the patient may ask about:

- Effectiveness
- Safety (including immunogenicity)
- Price
- Efficiency
- Responsibility for change
- Reason for change
- Possibility of avoiding therapeutic exchange
- Device

1. In all your experience of communication in the therapeutic exchange, which messages do patients receive best and which worst?
