# Supplementary material for: An Educational Digital Tool to Improve the Implementation of Switching to a Biosimilar (Rapid Switch Trainer): Tool Development and Validation Study
Source: JMIR Form Res. 2024 Nov 21;8:e56553. doi: 10.2196/56553 (PMC11612528; doi:10.2196/56553)
Supplement: Multimedia Appendix 2 [file formative-v8-e56553-s002.docx]

# Supplementary material

**Table S1.** Participants’ profiles.

| **Type** | **Gender** | **Specialty / Disease group** | **Region** |
| --- | --- | --- | --- |
| ***Interviews*** | | | |
| Clinician | Male | Dermatology | Valencia |
| Clinician | Male | Dermatology | Catalonia |
| Clinician | Male | Gastroenterology | Aragon |
| Clinician | Female | Rheumatology | Balearic Islands |
| Clinician | Female | Rheumatology | Andalusia |
| Clinician | Male | Rheumatology | Madrid |
| Clinician | Male | Rheumatology | Murcia |
| Nurse | Female | Dermatology | Canary Islands |
| Nurse | Female | Gastroenterology | Balearic Islands |
| Nurse | Female | Rheumatology | Andalusia |
| Nurse | Male | Rheumatology | Andalusia |
| ***Focus groups*** | | | |
| Clinician | Male | Dermatology | Catalonia |
| Clinician | Male | Rheumatology | Andalusia |
| Clinician | Male | Gastroenterology | Balearic Islands |
| Clinician | Female | Gastroenterology | Andalusia |
| Clinician | Male | Rheumatology | Murcia |
| Clinician | Male | Dermatology | Catalonia |
| Clinician | Male | Rheumatology | Catalonia |
| Nurse | Female | Gastroenterology | Castilla-Leon |
| Nurse | Female | Rheumatology | Andalusia |
| Nurse | Female | Rheumatology | Murcia |
| Nurse | Male | IMID | Canary Islands |
| Nurse | Female | Rheumatology | Catalonia |
| Nurse | Female | IMID | Andalusia |

**Table S2**. Arguments used by healthcare professionals to explain the change to a biosimilar.

| *“Similar medicine, equivalent molecule”*  *“Small differences that do not affect health status”*  *“Different molecule, not exactly the same medication”*  *“Same efficacy and safety as the reference”*  *“Same factoring quality, by reference laboratories”*  *“Just like a generic”*  *“They save money for the health system”*  *“Sometimes they are better than the previous because of changes in the mode of delivery”*  *“Same dosing and same follow-up as the previous treatment”*  *“Many studies have demonstrated their efficacy and safety”*  *“With the switch, we can treat more patients”*  *“We have to change for economic reasons”*  *“The new medication has been adjudicated to the hospital”*  *“Resources are limited, and more people can be treated with the new treatment.*  *“We contribute to the sustainability of the health system”*  *“There are different brands for the same molecule”*  *“The device is better”* |
| --- |

**Table S3**. Patients’ objections experienced by the healthcare professionals.

| *“The change is because is cheaper and cheaper is worse”*  *“The new treatment is less efficacious and safe”*  *“Why change if the treatment is working well?”*  *“May I go back to the previous treatment if this doesn’t work?”*  *“When will I notice the effect of the new treatment?”*  *“What can I do to avoid it?”*  [According to their healthcare professionals] The patient will experience nocebo if   - he/she leaves without answer to some doubts - has been using unreliable sources of information - the duration of the conversation was too short - they think cheaper means less efficacious - they had bad experiences with other medications - they do not trust the doctor/nurse |
| --- |

**Table S4.** Example of the feedback on an argument used to answer patients’ questions on biosimilars. In this case, the argument was charged as inadequate to promote trust.

| **Argument** | ***Feedback*** | ***Option(s)*** |
| --- | --- | --- |
| Biosimilars are cheaper | Biosimilars are indeed cheaper. However, basing the reason for the switch on this variable can lead the patient to feel that they are a "unit of healthcare expenditure" and that the savings occur independently of the quality of the healthcare they receive.  Including an explanation that the switch has no impact on efficacy/safety and is beneficial to the patient would help increase patient confidence. | There is no difference between a biosimilar and the reference drug regarding efficacy and safety. Biosimilars are a more efficient option, which could allow a greater number of patients to be treated and, in addition, to have access to other, more expensive, innovative therapies. |

**Table S5.** Technical information on the development of the APP.

| General specification of the project | Tasks prior to development. Include initial and follow-up meetings, technical and functional specifications of the Web APP, and the coordination and follow-up tasks of the work team. |
| --- | --- |
| Graphic design | Adapting the graphic proposal that Profarmaco2 will carry out/deliver. Programming automatically adapted to the different screen resolutions of the different mobile devices currently on the market (Smartphones, tablets, etc..) following the design guidelines of Web APP for Android, iPhone and iPad. |
| HTML5 programming | Programming of the Web APP following the specification and design. The web app was programmed in HTML5 standards for its subsequent adaptation to the different devices in which it is agreed to perform the distribution. |
| Programming server scripts | Programming in PHP and MySQL of the back office system where the contents obtained through JSON queries on the web server have been published. |
| HTML5 + CSS3 + JAVASCRIPT | For both the development and design of websites, it is necessary to write in certain languages during the layout. The choice of HTML5, CSS3, and JavaScript allowed us to offer and achieve a superior user experience. The design of websites in HTML5 gives us the possibility that the pages load much faster than in a site developed in previous versions of HTML. |
| Compatibility | All browsers: Firefox, Chrome, Explorer, Safari, Opera |
| Optimisation | Responsive Web Design technology that adapts the display of the web to different devices was used. |
