# Supplementary figures and images for: An Educational Digital Tool to Improve the Implementation of Switching to a Biosimilar (Rapid Switch Trainer): Tool Development and Validation Study
Source: JMIR Form Res. 2024 Nov 21;8:e56553. doi: 10.2196/56553 (PMC11612528; doi:10.2196/56553)

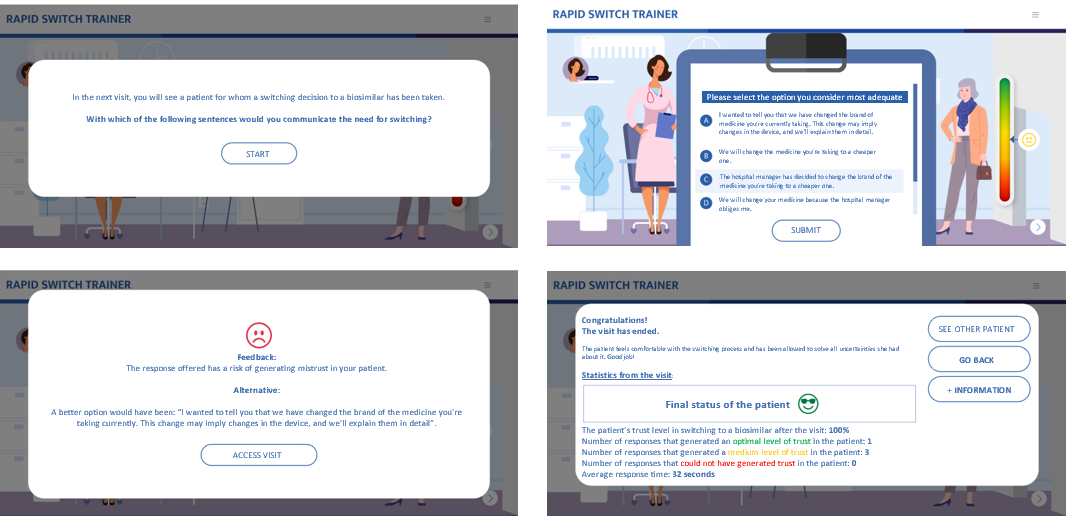

Supplement: Multimedia Appendix 3 [file formative-v8-e56553-s003.png]
