# Supplementary material for: An Educational Digital Tool to Improve the Implementation of Switching to a Biosimilar (Rapid Switch Trainer): Tool Development and Validation Study
Source: JMIR Form Res. 2024 Nov 21;8:e56553. doi: 10.2196/56553 (PMC11612528; doi:10.2196/56553)
Supplement: Multimedia Appendix 4 [file formative-v8-e56553-s004.docx]

EXAMPLES OF RAPID SWITCH TRAINER TOOL CONTENTS

1= Number of responses that have generated an optimal level of confidence in the patient.

0= Number of responses that have generated an average level of confidence in the patient.

-1= Number of responses that might not have generated confidence in the patient.

Objection 1: What is a biosimilar?

- A medicine that contains the same active substance as the biological medicine you were receiving. 1
- It is a medicine that contains the same active substance as the biological medicine you were receiving and has the same efficacy and safety. 1
- It is a drug that contains the same molecule as the biological medicine you have been receiving until now. 1
- It is a drug that contains the same molecule as the biological medicine you have been receiving so far with the same efficacy and safety. 1
- It is a drug that is essentially the same as the biological drug you were receiving. 0
- It is a drug that is highly similar to the biological drug you were receiving. -1
- It is a drug that replicates a drug that has lost its patent. -1
- It is a kind of generic and therefore cheaper than the original medicine. -1

Objection 3: How does the biosimilar act in my body?

- There is no difference in the way this medicine works in your body compared to the medicine you were receiving. 1
- It will act in the same way as the medicine you were receiving. 1
- The biosimilar medicine has been rigorously evaluated and shown to have the same mechanism of action, efficacy and safety as the medicine you were receiving, so it will work in the same way. 1
- The biosimilar medicine contains the same active substance as the medicine you were receiving, so its mechanism of action is the same, as well as in terms of efficacy and safety. 0
- Both have the same mechanism of action. -1
- In principle, they should act in the same way. -1
- You should not notice anything different, if you do notice a difference let us know as soon as possible. -1

Objection 9: Will the adverse effects improve with the switch to biosimilar?

- As the two drugs have the same active substance, they have the same safety profile and therefore no change is expected.1
- Safety and efficacy are expected to be equal.0
- You should not experience any difference in the efficacy and safety of the drug.0
- You are not expected to have any adverse effects other than those you may have experienced with your current treatment.0
- I cannot assure you that adverse effects will improve. -1
- It is possible, in medicine nothing can be said with absolute certainty. -1
- It does not have to. -1

Objection 15: What does it mean that biosimilars are more efficient?

- The fact that a biosimilar is more efficient means that more patients can access them with the same resources. 1
- That, by using a medicine with the same guarantees of efficacy and safety, we can treat more patients. 1
- Which has the same efficiency using fewer resources. 0
- That the relationship between efficiency and resources used is positive. 0
- That biosimilar medicines are cheaper. -1
- That generate savings favouring the sustainability of the Health System. -1
